# Two long-lasting human monoclonal antibodies cross-react with monkeypox virus A35 antigen

**DOI:** 10.1038/s41421-023-00556-w

**Published:** 2023-05-25

**Authors:** Bing Zhou, Haiyan Wang, Lin Cheng, Chengyan Zhao, Xinrong Zhou, Xuejiao Liao, Xiangyang Ge, Lei Liu, Xiaobo Lu, Bin Ju, Zheng Zhang

**Affiliations:** 1 Institute for Hepatology, National Clinical Research Center for Infectious Disease, Shenzhen Third People’s Hospital; The Second Affiliated Hospital, School of Medicine, Southern University of Science and Technology, Shenzhen, Guangdong China; 2 Infectious Disease and Liver Disease Department, The First Affiliated Hospital of Xinjiang Medical University, Urumqi, Xinjiang China; 3 Guangdong Key Laboratory for Anti-infection Drug Quality Evaluation, Shenzhen, Guangdong China; 4 Shenzhen Research Center for Communicable Disease Diagnosis and Treatment of Chinese Academy of Medical Science, Shenzhen, Guangdong China

**Keywords:** Immunology, Molecular biology

Dear Editor,

Human monkeypox has been a rare zoonotic disease caused by the monkeypox virus (MPXV) infection which is mainly reported in West and Central Africa in the past^1^. However, since the first confirmed case was found in the United Kingdom on May 7, 2022, there were over 87,000 confirmed cases worldwide until April, 2023 (https://worldhealthorg.shinyapps.io/mpx_global/). Human monkeypox seems to have become a multi-country outbreak disease and it was declared a Public Health Emergency of International Concern by the World Health Organization on July 23, 2022.

MPXV belongs to a member of Orthopoxvirus (OPXV) genus and is close to the infamous smallpox virus, yet causing relatively milder illness in humans^2^. MPXV infection causes 3.6% and 10.6% fatality in the West African clade and the Central African clade, respectively^3,4^. MPXV has a broad host range and many mammalian cells are susceptible to its infection^5^. Smallpox vaccines are hopeful to fight against MPXV, which could provide about 85% protection^6^, and could induce long-term humoral immunity for up to 75 years^7,8^. In this study, we evaluated the cross-recognization of this preexisting humoral immune response to MPXV including circulating polyclonal antibodies (pAbs) and memory B cells (MBCs), and revealed the key features of two isolated monoclonal antibodies (mAbs).

OPXV contains two kinds of virions, mature virion (MV) and enveloped virion (EV), carrying different surface antigens^9^. Taking vaccinia virus (VACV) as an example, several neutralizing antibody targets have been identified, such as L1, H3, A13, A17, and A27, and etc. on the MV and B5 and A33 on the EV^3^. We selected four representative homologous proteins of MPXV (H3 (equivalent to H3 in VACV) and A29 (A27 in VACV) on the MV; B6 (B5 in VACV) and A35 (A33 in VACV) on the EV)^10^, to detect the long-lasting antibody cross-binding to the MPXV in humans. After 1980, smallpox had been eradicated by the VACV vaccination^4^, and the vaccination program ceased. We randomly selected 211 plasma collected from individuals born before 1980 who visited the hospital due to other diseases rather than smallpox-related symptoms, and 27 plasma from individuals born after 1980 from the BioBank of Shenzhen Third People’s Hospital (Supplementary Table S1), and established a series of enzyme-linked immunosorbent assays (ELISAs) to evaluate the cross immune reaction.

Due to the lack of historic vaccination records, we could not confirm whether these people were immunized with relevant vaccines or not. Therefore, we first measured the antibody binding to A33 and A27 of VACV. As shown in Fig. 1a, the positive rate of A33-specific IgG was 32.2% (68/211) in people born before 1980. Fewer tested samples (11.4%, 24/211) contained anti-A27 IgG. None of 27 plasma from people born after 1980 bound to A33 and A27. Meanwhile, we measured the antibody response to other irrelevant viral antigens. Plasma from these two cohorts had comparable antibody levels to GP350 of Epstein-Barr virus and HA, NA, and NP of Influenza B virus (Supplementary Fig. S1). For the binding potency, the mean optical density (OD) value of anti-A33 IgG was significantly higher than that of anti-A27. Similar results were observed for A35 and A29 of MPXV. Sequence alignment partly explained the same trend between these homologous proteins sharing over 90% amino acid identity (Supplementary Fig. S2). It is important to note that the overall potency and positive rate of antibody binding to A35 and A29 was significantly lower than that to A33 and A27, respectively. This result, on the one hand, explained that the immunologic memory for MPXV was derived from the previous VACV vaccination. On the other hand, it indicated distinct immunogenicities between MPXV and VACV. Combined with the results of anti-B6 and anti-H3 IgGs, EV antigens (A35 and B6) induced a stronger or more durable antibody response than MV (A29 and H3). We also measured the antibody binding to A26, an MPXV-specific antigen without homologs in VACV^10^. Nearly no plasma bound the recombinant A26 protein with N-GST tag, except for one sample displaying a weak binding signal for some unknown reasons, which suggested that these people might not be exposed to MPXV. We further confirmed the binding activities of anti-VACV/MPXV IgG in subsequent follow-up plasma (Fig. 1b), suggesting that these circulating antibodies could last longer in humans. Due to the limited resource of live MPXV, we used live vaccinia virus as a model to assess the neutralizing antibodies (nAbs) in some available plasma containing anti-A33 and/or anti-A27 IgGs. As shown in Fig. 1c, 56.4% (44/78) of plasma samples could inhibit the infection of live vaccinia virus, with the maximum neutralization effect (Emax) of more than 50% at 1:20 dilution. The neutralizing activity of anti-A33 and anti-A27 double-positive plasma, especially the positive rate, was higher than that of single-antibody-positive samples, indicating that the combination of pAbs might contribute more to the neutralization against the live vaccinia virus in the focus reduction neutralization test (FRNT).

**Fig. 1:**
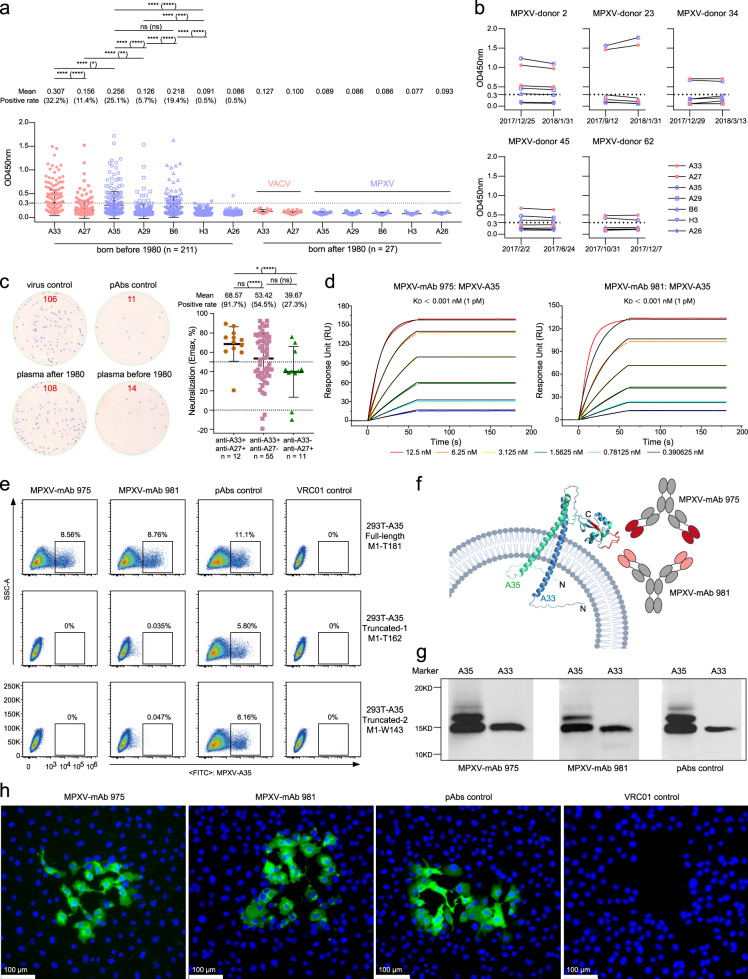
Long-term humoral immune responses and two human monoclonal antibodies cross-react with monkeypox virus. **a** ELISA screening of plasma IgG bound to A33 and A27 of VACV and A35, A29, B6, H3, and A26 of MPXV. All plasma were diluted at 1: 100. The positive cut-off was set as 0.3, which was more than 2-fold higher than background. **b** IgG binding ability to VACV and MPXV in plasma obtained from follow-up visits. **c** Neutralization results of available plasma (1:20 dilution) containing anti-A33 and/or anti-A27 IgG against live vaccinia virus by FRNT. Representative immune spots and counts were shown in left. Overall neutralization results were displayed in right. **d** Binding affinities of MPXV-mAb 975 and MPXV-mAb 981 to MPXV-A35 by SPR. **e** Epitope analysis of MPXV-mAb 975 and MPXV-mAb 981 recognizing to MPXV-A35. Full-length and C-terminal truncated MPXV-A35 were detected by flow cytometry. **f** Predicted models of MPXV-A35 (green) and VACV-A33 (blue) recognized by MPXV-mAb 975 and MPXV-mAb 981. Mainly targeted regions of MPXV-A35 and VACV-A33 were shown in red. Structures were prepared by PyMol. Membrane was created using BioRender. **g** Western blot analysis of MPXV-mAb 975 and MPXV-mAb 981 bound to MPXV-A35 and VACV-A33. **h** Immunofluorescence analysis of live vaccinia virus recognized by MPXV-mAb 975 and MPXV-mAb 981. Data in **a**–**d** were means of two independent experiments. Data in **a** and **c** were presented in mean ± standard deviation. Statistical significances were performed using two-tailed paired (**a**) or unpaired (**c**) Wilcoxon test for mean values and two-sided binomial test for positive rates using R 4.1.3. ****P < 0.0001; ***P < 0.001; **P < 0.01; *P < 0.05; ns not significant. Commercial pAbs were used as positive control. VRC01, an HIV-1 mAb, was used as negative control.

To explore another layer of long-term antibody memory, we tried to stain the MPXV-specific MBCs and identify mAbs from 2 donors, whose peripheral blood mononuclear cells were collected after 2020. The MPXV-A35 protein was used to sort antigen-specific MBCs with the phenotype of CD19^+^CD3^–^CD8^–^CD14^–^CD27^+^IgG^+^A35^+^ by flow cytometry (Supplementary Fig. S3). Using previously established platform^11^, we obtained two distinct human mAbs, MPXV-mAb 975 and MPXV-mAb 981, which showed strong binding capacities to MPXV-A35 and VACV-A33 in ELISA (Supplementary Fig. S4). Sequence analysis revealed that heavy chains of MPXV-mAb 975 and MPXV-mAb 981 were derived from *IGHV4-4* and *IGHV4-39* germline gene sequences, respectively, yet both of light chains belonged to *IGKV3-20*. The degrees of somatic hypermutation for MPXV-mAb 975 and MPXV-mAb 981 were 2.78% and 5.50% for heavy chains, and 2.62% and 1.50% for light chains, respectively. MPXV-mAb 975 and MPXV-mAb 981 mAbs possessed a 17-aa heavy chain complementarity determining region 3 (HCDR3) loop and a 9-aa kappa chain CDR3 (KCDR3) loop (Supplementary Table S2). In the surface plasmon resonance (SPR) assay, MPXV-mAb 975 and MPXV-mAb 981 bound to the MPXV-A35 with extremely high affinities (*K*_D_ < 1 pM) (Fig. 1d).

Denatured MPXV-A35 protein could be still recognized by MPXV-mAb 975 and MPXV-mAb 981 as well as its native form did, indicating that these two mAbs could target linear epitopes (Supplementary Fig. S5). To further narrow the recognition regions of MPXV-mAb 975 and MPXV-mAb 981, we constructed a full-length and several C-terminal truncated A35 variants and expressed them individually on the surface of 293T cells by transient transfection. Our flow cytometry analysis showed that MPXV-mAb 975 and MPXV-mAb 981 mainly recognized the C-terminal domain of A35 (163-TSDYQDSDVSQEVRKYFCT-181) (Fig. 1e). Using Alphafold2^12^, we predicted the structures of MPXV-A35 and VACV-A33 based on their amino acid sequences and displayed them on the membrane. MPXV-mAb 975 and MPXV-mAb 981 competed with each other in the competition ELISA (Supplementary Fig. S6) and targeted the overlapped region between MPXV-35 and VACV-A33 (Fig. 1f), which is supported by the sequence alignment (Supplementary Fig. S2). In this study, we also explored the potential applications of MPXV-mAb 975 and MPXV-mAb 981. The immunoblot analysis demonstrated that these two mAbs could detect the main bands of MPXV-A35 and VACV-A33, consistent with the commercial pAbs (Fig. 1g). Meanwhile, we measured the abilities of MPXV-mAb 975 and MPXV-mAb 981 to recognize the live vaccinia virus used in the immunofluorescence assay. As shown in Fig. 1h, the cellular localizations of A33 in the VACV-infected Vero E6 cells were clearly visualized by the recognization of MPXV-mAb 975 and MPXV-mAb 981. These results indicated that these two mAbs could be considered as good candidates for the detection of MPXV and VACV with potent binding affinities.

Obvious positive antibody responses occurred in donors born before 1980 rather than after 1980, strongly indicating that these positive responses were closely related to previous smallpox vaccination. Considering the waning immunity induced by the smallpox vaccine in individuals born before 1980 and the lack of vaccination in individuals born after 1980, the MPXV infection is still a serious challenge to the immune barrier in humans, especially for the high-risk population. This study provided direct and key evidence to support that previous smallpox vaccine-induced humoral immune responses including circulating plasma antibodies and virus-specific MBCs could persist for at least 40 years and could cross-react with the current concerned MPXV. We reported two human mAbs, potently binding MPXV-A35 and effectively recognizing live vaccinia virus. Previous research showed that optimal protection against the infection of VACV required a mixture of mAbs targeting several membrane proteins on the EV and MV^13^. The live vaccinia virus used in the neutralization assay of this study did not distinguish EVs from MVs. Therefore, we did not confirm whether these two mAbs targeting the A33 on the EV could participate in the process of blocking infections. According to the neutralization results of polyclonal plasma, there indeed exists a combination of monoclonal nAbs in some people. In future, we will isolate more anti-A33/A35 mAbs and other mAbs targeting distinct membrane proteins including A27/A29 on the MV, and comprehensively evaluate their functions and potential clinical applications in therapeutic antibody research.

## Supplementary information

supplementary information

Ethic approval
