# Supplementary material for: Two long-lasting human monoclonal antibodies cross-react with monkeypox virus A35 antigen
Source: Cell Discov. 2023 May 25;9:50. doi: 10.1038/s41421-023-00556-w (PMC10213010; doi:10.1038/s41421-023-00556-w)
Supplement: Supplementary file 1 — supplementary information [file 41421_2023_556_MOESM1_ESM.pdf]

Supplementary information

Materials and Methods

Study approval and biological samples

This study was approved by the Ethics Committee of Shenzhen Third People's Hospital, China (approval number: 2021-030). The participants had provided written informed consent for sample collection and subsequent analysis. All plasma and peripheral blood mononuclear cells (PBMCs) samples were stored at the BioBank of Shenzhen Third People's Hospital. All plasma samples were stored at -80 °C and heat-inactivated at 56 °C for 1 h before use. PBMCs were maintained in freezing medium and stored in liquid nitrogen before use.

Enzyme linked immunosorbent assay (ELISA)

The native recombinant proteins of MPXV, VACV, Epstein-Barr virus, and Influenza B virus (Sino Biological and AntibodySystem) or denatured by treatment with the denaturing buffer (New England Biolabs) at 95 °C for 10 mins were coated into 96-well plates at 4 °C overnight. The plates were washed with PBST buffer and blocked with 5% skim milk and 2% bovine albumin in PBS at RT for 1 h. Diluted plasma samples and monoclonal antibodies (mAbs) were added into wells and incubated at 37 °C for 1 h. The plates were washed and HRP conjugated goat anti human IgG antibodies (ZSGB-BIO) were added and then incubated at 37 °C for 30 mins (plasma) and for 1 h (mAbs). Finally, the TMB substrate (Sangon Biotech) was added and incubated at RT for 5 mins (plasma) and for 20 mins (mAbs) and the reaction was stopped by 2M H_2SO_4. The readout was detected at a wave length of 450 nm. The 50% effective concentration (EC_{50}) values of tested mAbs were calculated using GraphPad Prism 8 software by log (agonist) vs. response -- Variable slope (four parameters) model.

Competition ELISA

The 96-well plates were coated with MPXV A35 protein (Sino Biological) at 4 °C overnight. The plates were washed with PBST buffer and blocked with 5% skim milk and 2% bovine albumin in PBS at RT for 1 h. Serially diluted mAbs were mixed with HRP (Abcam) conjugated MPXV-mAb 975 or MPXV-mAb 981 in

equal volume and then added to the plates and incubated at 37 °C for 1 h. Finally, the TMB substrate (Sangon Biotech) was added and incubated at RT for 20 mins and the reaction was stopped by 2M H_2SO_4. The readout was detected at a wave length of 450 nm. The competitive effect was determined by comparing the ratio of different mAbs to the negative control mAb (VRC01, an HIV-1 mAb).

Neutralization assay

For live VACV preparations. Vero E6 cells in T75 were infected at a multiplicity of infection (MOI) of 0.1. Cells were harvested at Day 2, and virus was isolated by rapidly freeze-thawing the cell pellet three times in a volume of 5 mL of DMEM supplemented with 2% heat-inactivated FBS (D-2). Cell debris was removed by centrifugation at 800 g for 5 mins. The virus containing supernatant was aliquoted and stored at -80 °C.

To test the neutralization activity of the heat inactivated human plasma, Vero E6 cells were seeded at 1.5×10^4 cells/well into 96-well plates and used the following day. Plasma samples 10-fold diluted in D-2 medium with 2% sterile guinea pig complement (Beijing Bersee Science and Technology Co.,Ltd) were mixed with equal volume of diluted live VACV and then incubated at 37 °C for 1 h. Medium from 96-well plates was aspirated, and plasma-virus mixture was added and allowed to adsorb at 37 °C for 1 h. Cells were rinsed with warm PBS, overlaid with D-2 medium, and the plates were incubated at 37 °C for 8 h. Then cells were fixed with 4% paraformaldehyde solution, permeabilized with Perm/Wash buffer (BD Biosciences) containing 0.1% Triton X-100, incubated with the HRP-conjugated anti-VACV polyclonal antibodies (Invitrogen) at 4 °C overnight. The reactions were developed with KPL TrueBlue Peroxidase substrates (Seracare Life Sciences). The numbers of VACV foci were calculated using an EliSpot reader (Cellular Technology Ltd).

Isolation of mAbs from MPXV-donor 3 and MPXV-donor 42

Thawed PBMCs were stained with an antibody cocktail including CD19-PE-Cy7, CD3-Pacific Blue, CD8-Pacific Blue, CD14-Pacific Blue, CD27-APC-H7, and IgG-FITC (BD Biosciences) to gate IgG+ memory B cells. MPXV-A35 with a C-

His tag (Sino Biological) was used as a probe to sort antigen-specific single cells. To exclude the nonspecific staining, two anti-His secondary antibodies labeled with APC and PE (Abcam) were both used to recognize the MPXV-A35 bait. The flow cytometric data were acquired on BD FACSymphony S6 (BD Biosciences) and analyzed using FlowJo software (TreeStar). Single B cells were sorted into 96-well PCR plates containing lysis buffer followed by RT-PCR and nested PCR to amplify variable regions of heavy- and light-chain. Variable genes were sequenced by Sangon Biotech, synthesized by GenScript, and then separately cloned into full-length IgG1 heavy and light chain expression vectors. Monoclonal antibodies were expressed by co-transfection of 293 F cells with paired heavy- and light-chain plasmids and purified from the culture supernatants using protein A column (GenScript).

Binding affinity analysis by surface plasmon resonance (SPR)

The binding assays of mAbs to the MPXV-A35 protein (Sino Biological) were performed using the Biacore 8K system (GE Healthcare). Specifically, one flow cell of the CM5 sensor chips were covalently coated with MPXV-A35 in 10 mM sodium acetate buffer (pH 5.0) for a final RU (response units) around 250, whereas the other flow cell was left uncoated and blocked as a control. All the assays were run at a flow rate of 30 μL/min in HBS-EP buffer (10 mM HEPES pH 7.4, 150 mM NaCl, 3 mM EDTA, and 0.05% Tween-20). Serially diluted antibodies were injected for 60 s respectively and the resulting data were fit in a 1:1 binding model with Biacore Evaluation software (GE Healthcare). Every measurement was performed two times and the individual values were used to produce the mean affinity constant.

Epitope analysis by flow cytometry

The 293 T cells transfected by full-length or truncated MPXV-A35 expression vectors were stained with the LIVE/DEAD Fixable Dead Cell Stain reagent (Thermo Scientific) to exclude dead cells. Cells were then incubated with MPXV-mAb 975, MPXV-mAb 981, VRC01, or anti-MPXV A35 polyclonal antibodies (Antibodysystem), followed by stained with Alexa Fluor 488-conjugated secondary antibody (Invitrogen). After washing, cells were

resuspended and subjected to acquisition with the FACSymphony S6 (BD Biosciences). Data were analyzed with FlowJo software (TreeStar).

Western blot assay (WB)

A35 of MPXV and A33 of VACV (Sino Biological) were loaded onto 15% sodium dodecyl sulphate polyacrylamide gel electrophoresis (SDS-PAGE), transferred to polyvinylidene difluoride (PVDF) membrane using the Mini-PROTEAN Tetra System (Bio-Rad). After blocking with 5% skim milk for 1 h at RT, membranes were incubated with MPXV-mAb 975, MPXV-mAb 981, or anti-MPXV A35 polyclonal antibodies (Antibodysystem) overnight at 4 °C, followed by incubation with HRP-conjugated Goat anti-Human IgG (ZSGB-BIO) or Goat anti-Rabbit IgG (H+L) (TransGen Biotech) for 1 h at RT. The proteins were visualized with Chemiluminescent Substrate (Thermo) and a ChemiDoc MP Imaging System (Bio-Rad).

Immunofluorescence assay (IFA)

Vero E6 cells were infected with vaccinia live viruses at a MOI of 0.002 for 1 h, then washed and cultured in the dulbecco's modified eagle medium (DMEM) containing 2% FBS. After 14 h, cells were fixed with 4% paraformaldehyde for 30 mins at RT, permeabilized with Perm/Wash (BD Biosciences) containing 0.15% Triton X-100 for 10 mins at RT. After washing, cells were stained with MPXV-mAb 975, MPXV-mAb 981, VRC01, or anti-VACV pAbs (Invitrogen) at 4 °C overnight, followed by staining with Alexa Fluor 488-conjugated secondary antibody (Invitrogen) for 1 h at RT. After washing, cells were incubated with Hoechst 33342 (Life Technologies) for 20 mins at RT. Microscopic images were obtained under a digital inverted microscope EVOS (Life technologies).

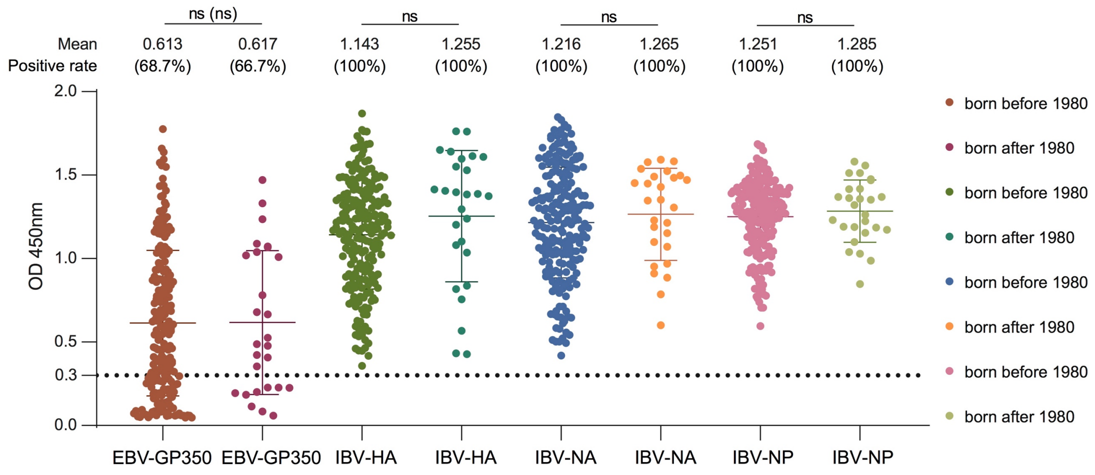

Supplementary Fig. S1. ELISA detection of plasma IgG binding to GP350 of Epstein-Barr virus (EBV) and HA, NA, and NP of Influenza B virus (IBV) in 211 individuals born before 1980 and 27 individuals born after 1980. The recombinant EBV GP350 (strain B95-8) and IBV HA (strain B/Austria/1359417/2021), NA (strain B/Washington/02/2019), and NP (strain B/Austria/1359417/2021) proteins were purchased from Sino Biological. All plasma samples were tested at the dilution of 1:100. The data were means of two independent experiments and presented in mean values ± SD. The mean, positive rate, and significance of difference were labelled on top. Statistical significance was performed using two-tailed unpaired Mann-Whitney test for mean values using the GraphPad Prism 8 software or two-sided binomial test for positive rates using the R 4.1.3 software. ns, not significant.

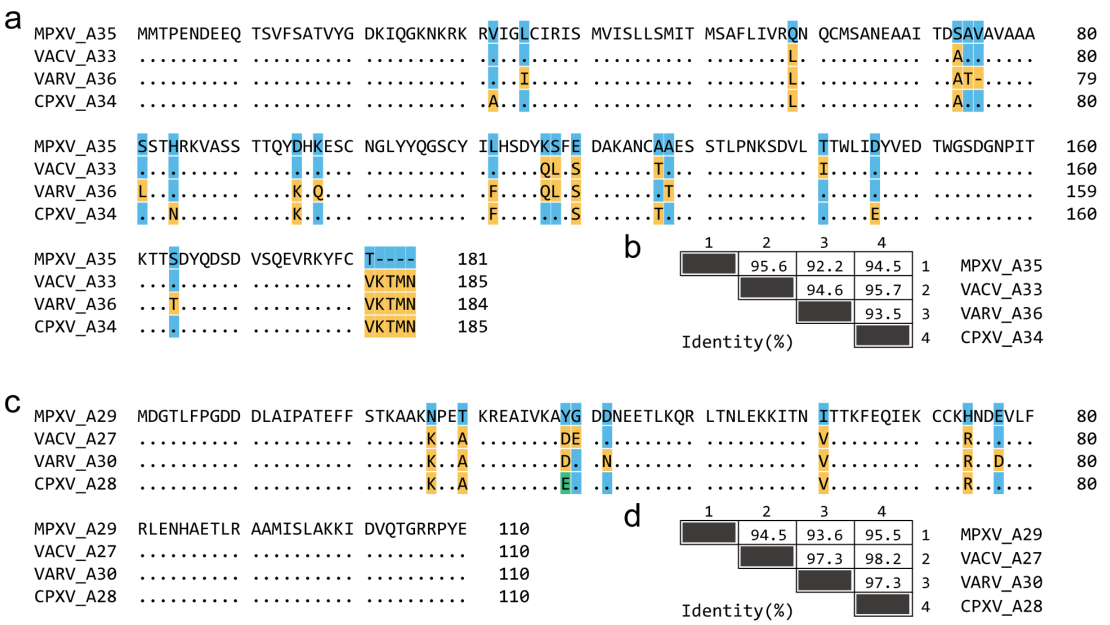

Supplementary Fig. S2. Amino acid sequence alignment and identity analysis among A35 or A29 of MPXV, A33 or A27 of VACV, A36 or A30 of VARV, and A34 or A28 of CPXV.

(a and c) Sequence alignment of MPXV-A35 or MPXV-A29 homologous proteins in four kinds of OPXV (MPXV: NC_003310.1, VACV: AY243312.1, VARV: X69198.1, CPXV: DQ437593.1). Amino acid residues consistent with MPXV-A35 or MPXV-A29 were marked in blue. Mutations in VACV, VARV, or CPXV were marked in yellow or green. **(b and d)** Pairwise identity (%) of aligned MPXV-A35 or MPXV-A29 homologous proteins of MPXV, VACV, VARV, and CPXV.

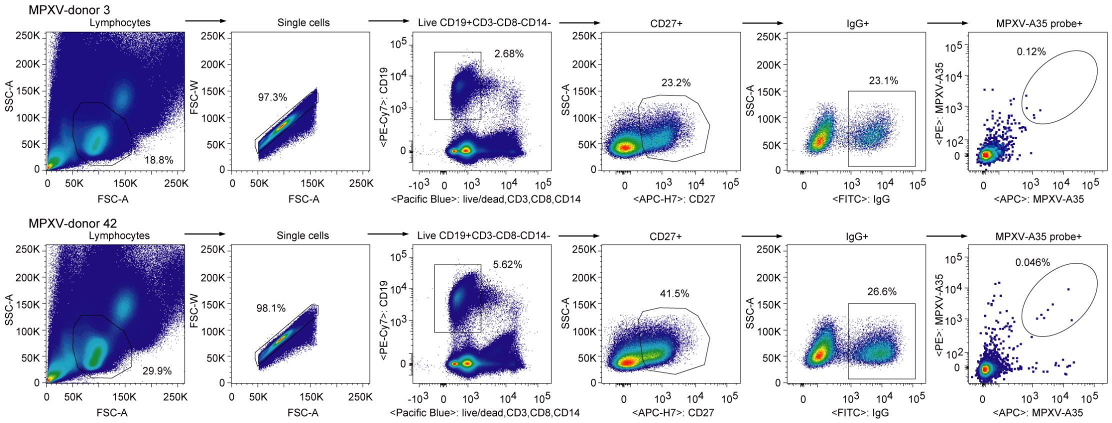

Supplementary Fig. S3. The isolation of two MPXV-A35-specific monoclonal antibodies.

Single cell sorting of MPXV-A35-specific MBCs by flow cytometry. The gating strategy was $CD19^{+}CD3^{-}CD8^{-}CD14^{-}CD27^{+}IgG^{+}A35^{+}$.

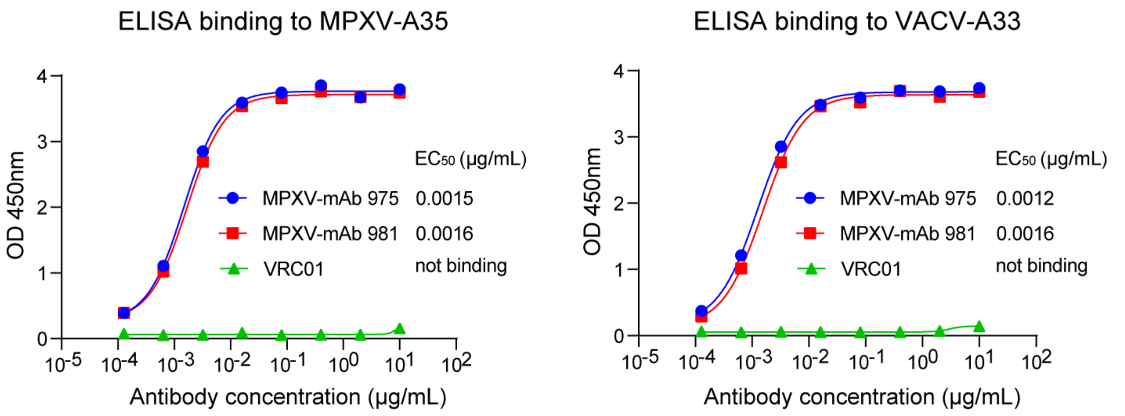

Supplementary Fig. S4. ELISA binding of MPXV-mAb 975 and MPXV-mAb 981 to the MPXV-A35 or VACV-A33 protein.

VRC01 was a known HIV-1-specific antibody as a negative control. The 50% effective concentration (EC_{50}) values were calculated using GraphPad Prism 9 software by log (agonist) vs. response -- Variable slope (four parameters) model. The data of EC_{50} were means of two independent experiments.

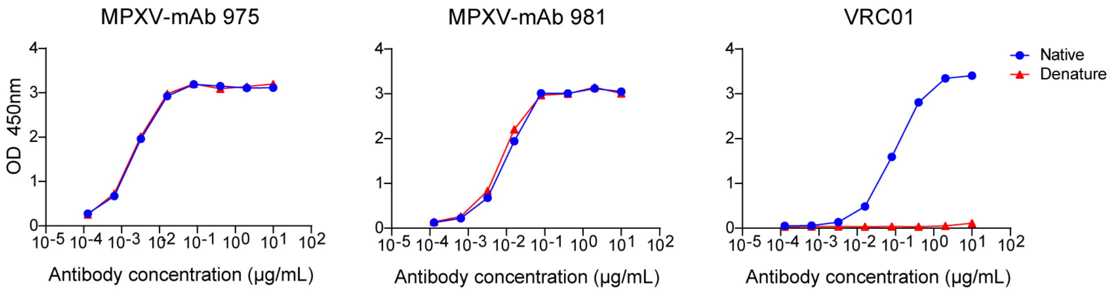

Supplementary Fig. S5. ELISA binding of MPXV-mAb 975 and MPXV-mAb 981 to native and denatured MPXV-A35 proteins.

VRC01 recognizing the conformational epitopes of HIV-1 GP140 was served as the positive control to prove that the denaturing process was efficient.

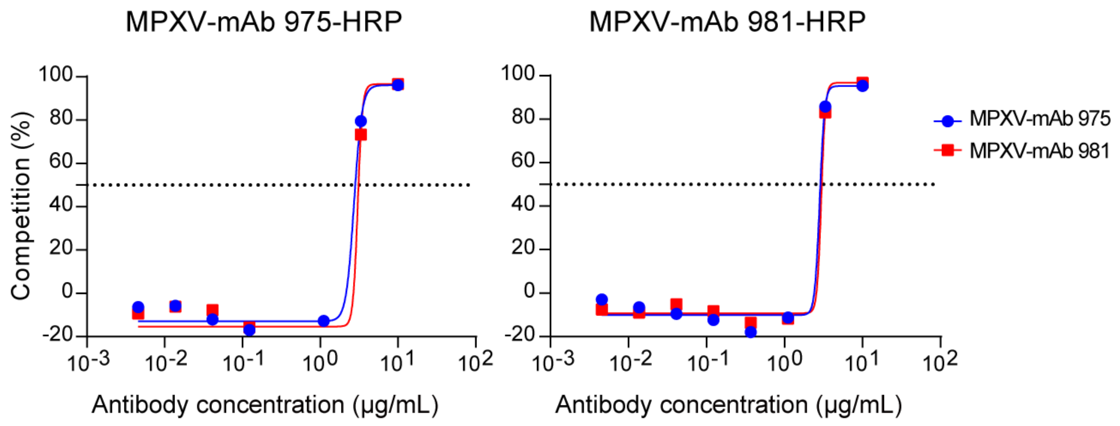

Supplementary Fig. S6. Competition ELISA between MPXV-mAb 975 and MPXV-mAb 981.

MPXV-mAb 975 and MPXV-mAb 981 were used as HRP-conjugated antibodies respectively.

Supplementary Table S1. Demographic characteristics of 211 individuals born before 1980 and 27 individuals born after 1980 involved in this study.

	Born before 1980 (n=211)	Born after 1980 (n=27)
Male (n, %)	156 (73.93%)	24 (88.89%)
Age (median, range)	50 (42-82)	20 (14-25)
Date of brith (median, range)	1969 (1936-1976)	2000 (1995-2003)
Sampling time (median, range)	2019 (2017-2022)	2020 (2017-2020)

Supplementary Table S2. Gene analysis of mAbs isolated from MPXV-donor 3 and MPXV-donor 42.

mAbs	Heavy chain			Light chain		
	IGHV	CDR3 (aa)	SHM (%)	IGKV	CDR3 (aa)	SHM (%)
MPXV-mAb 975	4-4	17	2.78	3-20	9	2.62
MPXV-mAb 981	4-39	17	5.50	3-20	9	1.50

The program IMGT/V-QUEST was applied to analyze the gene germline, the amino acid length of complementarity determining region 3 (CDR3), and the degree of somatic hypermutation (SHM). The SHM frequency was calculated from the mutated nucleotides.
