# Supplementary material for: Two long-lasting human monoclonal antibodies cross-react with monkeypox virus A35 antigen
Source: Cell Discov. 2023 May 25;9:50. doi: 10.1038/s41421-023-00556-w (PMC10213010; doi:10.1038/s41421-023-00556-w)
Supplement: Supplementary file 2 — Ethic approval [file 41421_2023_556_MOESM2_ESM.pdf]

目录

2022 年度生物样本库伦理跟踪审查意见函 1

2021 年度生物样本库伦理初始审查批件 4

深圳市第三人民医院伦理委员会科研项目伦理审查意见函

批件号：深圳三院伦审科研字[2021-030-06]号

<table>
<tr><td>项目名称</td><td colspan="4">深圳市第三人民医院生物样本库</td></tr>
<tr><td>申请类型</td><td colspan="4">院内课题</td></tr>
<tr><td>申请科室</td><td colspan="4">肝病研究所</td></tr>
<tr><td>申请人</td><td colspan="4">张政</td></tr>
<tr><td>审查文件</td><td colspan="4">1. 科研项目年度/定期跟踪审查申请表
2. 深圳市第三人民医院生物样本库年度运行情况总结
3. 科研项目年度/定期跟踪审查超期说明
4. 科研项目年度/定期跟踪审查超期说明（修改后）
5. 知情同意书（版本号：SZTPH-BRC-V 4.0，版本日期：2022-08-04）
6. 生物样本库保藏方案（版本号：V 3.0 版本日期：2022-07-15）</td></tr>
<tr><td rowspan="6">审查方式:
简易审查</td><td>审查类别</td><td>年度/定期跟踪审查</td><td>审查日期</td><td>2022年8月9日</td></tr>
<tr><td>评审委员</td><td>魏兰兰，廖子锐</td><td>审查地点</td><td>深圳市第三人民医院</td></tr>
<tr><td colspan="2">同意_1_票</td><td colspan="2">必要的修改后同意_1_票</td></tr>
<tr><td colspan="2">修改后再审_/_票</td><td colspan="2">不同意_/_票</td></tr>
<tr><td colspan="4">终止或者暂停已同意的研究_/_票</td></tr>
<tr><td>审查意见</td><td colspan="3">修改后同意</td></tr>
<tr><td rowspan="7">审查方式:
会议审查</td><td>审查类别</td><td>年度/定期跟踪审查</td><td>审查日期</td><td>2022年9月6日</td></tr>
<tr><td>评审委员</td><td colspan="3">刘威龙、付亮、刘厚明、朱质斌、安江宏、吴强、李巍、魏兰兰、巫韦奇、廖子锐</td></tr>
<tr><td>审查地点</td><td colspan="3">深圳市第三人民医院</td></tr>
<tr><td colspan="2">同意_10_票</td><td colspan="2">必要的修改后同意_/_票</td></tr>
<tr><td colspan="2">修改后再审_/_票</td><td colspan="2">不同意_/_票</td></tr>
<tr><td colspan="4">终止或者暂停已同意的研究_/_票</td></tr>
<tr><td>审查意见</td><td colspan="3">同意</td></tr>
<tr><td>批准文件</td><td colspan="4">1. 科研项目年度/定期跟踪审查申请表
2. 深圳市第三人民医院生物样本库年度运行情况总结
3. 科研项目年度/定期跟踪审查超期说明（修改后）
4. 知情同意书（版本号：SZTPH-BRC-V 4.0，版本日期：2022-08-04）
5. 生物样本库保藏方案（版本号：V 3.0 版本日期：2022-07-15）</td></tr>
<tr><td colspan="5">根据国家食品药品监督管理总局最新“药物临床试验质量管理规范”、“医疗器械临床试验规定”、“药物临床试验伦理审查工作指导原则”，卫生部“涉及人的生物医学研究伦理审查办法试行”，国家中医药管理局“中医药临床研究伦理审查管理规范”，WMA《赫尔辛基</td></tr>
</table>

宣言》和 CIOMS《人体生物医学研究国际道德指南》等要求，经本伦理委员会审查，意见如下：同意研究继续进行。

注意事项：
1、请遵循 GCP 原则、遵循伦理委员会同意的方案开展临床研究保护受试者的健康和权利。
2、本专项研究涉及人类遗传资源采集、保藏、利用、对外提供等，须遵照《中华人民共和国人类遗传资源管理条例》相关规定执行。
3、对研究方案、知情同意书、招募材料等的任何修改请提交修正案审查申请。
4、发生 SAE 请及时提交严重不良事件报告。
5、如有不依从/违背方案的情况请及时提交违背方案报告。
6、请根据年度/定期跟踪审查频率及时提交研究进展报告。
7、暂停或终止临床研究请及时提交暂停/终止研究报告。
8、完成临床研究请提交结题报告。

**年度/定期跟踪审查频率**	12 个月	**批件有效期**	2023 年 9 月 5 日（请在批件到期前 1 个月提交跟踪审查申请）
**主任委员（或被授权者）签字：** 张国良 日期：2022 年 9 月 6 日		**深圳市第三人民医院科研伦理委员会（盖章）**	

声明：本伦理委员会的职责、人员组成、运行和记录遵循 ICH-GCP、GCP，符合赫尔辛基宣言的原则，并遵守中国相关法律和法规的规定。

地址：深圳市龙岗区布澜路 29 号　　电话：0755-61222333-6539

深圳市第三人民医院医学伦理委员会审查会议签字页

（科研伦理委员会）

	序号	任职	姓名	性别	专业	签名	日期
科研伦理委员会成员	1	主任委员	张国良	男	感染病学	请假	2022-9-6
	2	副主任委员	朱国峰	男	分子遗传学	请假	2022-9-6
	3	委员	刘威龙	男	动物医学	出席	2022-9-6
	4	委员	付亮	男	结核病学	出席	2022-9-6
	5	委员	刘厚明	男	临床检验诊断学	出席	2022-9-6
	6	委员	朱质斌	男	传染病	出席	2022-9-6
	7	委员	安江宏	男	肿瘤学	出席	2022-9-6
	8	委员	吴强	男	麻醉学	出席	2022-9-6
	9	委员	李巍	男	药学	出席	2022-9-6
	10	委员	刘东京	女	生物医学工程	出席	2022-9-6
	11	委员	魏兰兰	女	病原微生物学	出席	2022-9-6
	12	委员	巫韦奇	男	法学	出席	2022-9-6
	13	委员	廖子锐	男	社会医学与卫生事业管理	出席	2022-9-6
	14	秘书	韩雨	女	社会医学与卫生事业管理	出席	2022-9-6

承诺： 作为深圳市第三人民医院伦理委员会的成员，我将对我所审阅临床研究资料、会议讨论结果和相关内容进行保密。遵守国家法律法律，遵守 GCP 规范，遵守医学伦理委员会管理制度，行使伦理委员会的职责，最大限度保护受试者的个人利益不受侵害。

深圳市第三人民医院

科研伦理委员会

附件 10：

科研立项资料形式审查表

递交资料（请研究者勾选）	审查结果（伦理秘书勾选）
形式审查表☑	☐项目负责人签名
项目方案☑<br>版本号：SZTPH-BB-V 02<br>版本日期：2021 年 06 月 01 日	项目方案：☐有，☐无<br>注明版本号：☐有，☐无<br>注明版本日期：☐有，☐无
知情同意书☑<br>豁免知情同意书☐<br>知情同意是否合规范☑是，☐否（参考 P10-11 须知九）<br>版本号：SZTPH-BB-V 02<br>版本日期：2021 年 06 月 30 日	知情同意是否合规范☐是，☐否<br>注明版本号：☐有，☐无<br>注明版本日期：☐有，☐无
研究者履历☑	研究者履历：☐有，☐无<br>填写及签字完整：☐是，☐否
研究经济利益声明☑	研究经济利益声明：☐有，☐无<br>填写及签字完整：☐是，☐否
后附装订标准<br>资料拉杆夹、文件盒装订☑<br>侧面标签☑	资料拉杆夹、文件盒装订☐<br>侧面标签☐
以上内容如不合格，项目退回，请研究者重新准备。	
汇报 PPT☑	汇报 PPT☐：☐有，☐无
是否组长单位：☐是，☑否<br>是否提供组长单位批准文件及其他意见：☐是，☑否	是否组长单位：☐是，☐否<br>是否提供组长单位批准文件及其他意见：☐是，☐否
如有，请勾选：<br>招募广告☐<br>保险证明☐<br>受试者日志☐<br>药物说明书☐<br>研究病历☐<br>其他：样本库成立相关院级文件 ☑<br>项目组成员名单 ☑	
项目来源：院内自筹<br>合作单位：无<br>科研立项证明：有	按科教科要求提供，原件退还研究者。<br>记录《科研立项伦理审查批准文件》-“递交资料”中。
递交人（研究者代表）签字：[signature]<br>时间：2021.6.30	科研伦理委员会盖章<br>提供结论：同意会议审查<br>时间：<br>经办人 [signature]<br>2021年07月06日

扫描全能王 创建

深圳市第三人民医院伦理委员会科研项目伦理审核批准文件

批件号：深圳三院伦审科研字[2021-030]号

**项目名称**	感染性疾病生物样本库标准化建设及管理		
**申请类型**	院内		
**申请专业**	肝病研究所	**申请人**	张政
**审查方式**	会议审查	**会议审查时间**	2021年07月15日
**审查内容**	1.科教科立项证明（退还研究者存档）；<br>2.研究方案（版本号：SZTPH-BB-V 02，日期：2021年06月01日）；<br>3.知情同意书（版本号：SZTPH-BB-V 02，日期：2021年06月30日）；<br>5.研究经济利益声明；<br>4.研究者履历（张政）；<br>6.汇报PPT		
**审 查 意 见**	1. 根据国家食品药品监督管理总局最新“药物临床试验质量管理规范”、“医疗器械临床试验规定”、“药物临床试验伦理审查工作指导原则”，卫生部“涉及人的生物医学研究伦理审查办法试行”，国家中医药管理局“中医药临床研究伦理审查管理规范”，WMA《赫尔辛基宣言》和CIOMS《人体生物医学研究国际道德指南》等要求，经本伦理委员会审查，意见如下：<br>经本伦理委员会审查，意见如下：<br>☑同意<br>研究方案（版本号：SZTPH-BB-V 02，日期：2021年06月01日）<br>知情同意书（版本号：SZTPH-BB-V 02，日期：2021年06月30日）<br>☐必要的修改后同意<br>☐不同意<br>☐终止或暂停已同意的研究<br>2. 该研究进行过程中将接受伦理委员会的跟踪审查？ ☑是 ☐否<br>3. 跟踪审查频率为：☐3个月 ☐6个月 ☑12个月<br>4. 批件有效期：2022年07月16日。		
	深圳市第三人民医院科研伦理委员会<br>主任/副主任委员（签名）：<br>2021年07月16日		

注意事项（请仔细阅读）：

1. 发生严重不良事件及影响研究风险受益比的非预期事件，须及时报告本伦理委员会；

本院发生SAE请在获知后24小时内报告伦理委员会

联系方式：

地址：深圳市龙岗区布吉镇布澜路29号李朗出口　　　　邮编：518112

扫描全能王 创建

电话：0755-61222333-6665　　　　　　　　　　　　　　　　　　邮箱：224131788@qq.com

2. 本项临床研究应当在伦理委员批准起1年内实施。逾期未实施的，本批件自行废止；

3. 暂停/提前终止临床研究，请及时通知伦理委员会；

4. 对已批准的研究方案、知情同意书等材料的任何修改及主要研究者更换等，须及时通知本伦理委员会重新审查，获得批准后执行；

5. 完成临床研究，须提交结题报告供伦理委员会审查。

扫描全能王 创建

深圳市第三人民医院医学伦理委员会审查会议签字页

（科研伦理委员会）

	序号	任职	姓名	性别	专业	签名	日期
科研伦理委员会成员	1	主任委员	何清	男	传染病学		2021-07-15
	2	副主任委员	黄婷	女	超声影像学		2021-07-15
	3	委员	刘东京	女	生物医学工程专业	请 假	2021-07-15
	4	委员	叶涛生	男	结核病学		2021-07-15
	5	委员	何云	女	感染病学		2021-07-15
	6	委员	张明霞	女	免疫学	请 假	2021-07-15
	7	委员	舒丹	女	感染病学		2021-07-15
	8	委员	乔坤	男	外科学		2021-07-15
	9	委员	张国良	男	感染病学		2021-07-15
	10	委员	黄佳	男	临床医学		2021-07-15
	11	委员	周建忠	男	法学		2021-07-15
	12	委员	彭四根	男	法学		2021-07-15
	13	委员	李昱熹	女	护理学	请 假	2021-07-15
	14	秘书	黄芳	女	临床医学		2021-07-15

承诺： 作为深圳市第三人民医院伦理委员会的成员，我将对我所审阅临床研究资料、会议讨论结果和相关内容进行保密。遵守国家法律法律，遵守 GCP 规范，遵守医学伦理委员会管理制度，行使伦理委员会的职责，最大限度保护受试者的个人利益不受侵害。

深圳市第三人民医院

科研伦理委员会

扫描全能王 创建
